# A Formula to Calculate the Threshold for Radiotherapy Targets on PET Images: Simulation Study

**DOI:** 10.3389/fonc.2020.550096

**Published:** 2020-10-21

**Authors:** Jianhua Geng, Fei Luo, Jiahe Tian, Jinming Zhang, Xiaojun Zhang, Baolin Qu, Yingmao Chen

**Affiliations:** ^1^Department of Nuclear Medicine, National Cancer Center/National Clinical Research Center for Cancer/Cancer Hospital, Chinese Academy of Medical Sciences and Peking Union Medical College, Beijing, China; ^2^Department of Radiation Oncology, National Cancer Center/National Clinical Research Center for Cancer/Cancer Hospital, Chinese Academy of Medical Sciences and Peking Union Medical College, Beijing, China; ^3^Department of Nuclear Medicine, Chinese PLA General Hospital, Beijing, China; ^4^Department of Radiotherapy, Chinese PLA General Hospital, Beijing, China

**Keywords:** positron-emission tomography, radiotherapy, threshold, phantom, imaging

## Abstract

**Background:**

Positron emission tomography (PET) images are being applied for defining radiotherapy targets. However, a recognized method for defining radiotherapy targets is lacking. We investigate the threshold to outline the radiotherapy target of a tumor on PET images and its influencing factors, and then expressed it by formula.

**Methods:**

PET imaging for spherical tumors with a different tumor diameter (D), under different system resolutions [full width at half maximum (FWHM)], in different backgrounds with different pixel sizes, was simulated. PET images were analyzed to determine the relationship between the threshold and the factors mentioned above. Finally, the simulation results were verified by phantom experiments.

**Results:**

The threshold decreased sharply with D for D < 2 FWHM, reached the minimum of 31% at D = 2 FWHM and then increased slowly, and it tended to constant for D > 8 FWHM. The threshold decreased with FWHM for FWHM < D/2, reached a minimum at FWHM = D/2, and then increased. The threshold increased with pixel size for D ≤ FWHM and decreased for D > FWHM. The threshold was independent of the background. The relationship between the threshold and its influencing factors was expressed as a formula. The results of the phantom verification indicated that the error of the target volume delineation that was calculated by the formula was less than 9%.

**Conclusions:**

The threshold changes with tumor size, resolution of the PET system and pixel size according to certain rules. The formula to calculate the threshold could provide a method to estimate threshold to outline the radiotherapy target (tumor).

## Introduction

Positron emission tomography (PET) images can reflect quantitatively the biologic metabolism of tumor. Hence, fluorodeoxyglucose (FDG)-PET imaging has been used more frequently in defining biologic targets for radiotherapy in recent years (1–4). Several authors have declared that a metabolic tumor volume (MTV) based on the FDG accumulated fraction of the entire tumor is a more sensitive and reliable indicator for therapeutic monitoring in comparison with that using other biomarkers. Nevertheless, because of the influence of the partial volume effect (PVE), tumor boundaries on PET images are blurred and the exact size of a tumor cannot be defined precisely. How to determine tumor boundaries and delineate the biologic target of a tumor has been an important focus of research (5–21). Bearing this in mind, we undertook a computer-simulation study on various imaging parameters that might be encountered when conducting imaging with different device performances under various clinical situations.

The methods reported for defining tumor boundaries vary considerably (22–25). The main reasons for this variation are differences in performance of the imaging devices, reconstruction algorithms and settings, as well as the target volume itself. Briefly, the spatial resolution, pixel size of reconstructed images, tumor size, cutoff value (threshold) for the tumor edge, FDG uptake, and FDG-avid tumor mass and its background are among the major factors to be considered if MTV is to be delineated precisely. The complexity of the clinical scenario and difficulty in normalizing those factors in real cases means that an ideal way to test those variables is through computer simulation. In this way, a predefined “tumor” can be produced in computer space and, *via* selection of the items mentioned above in the simulation; the effects of each variable on the assessed MTV against the “virtual” tumor can be clarified and compared. With an appropriate design, the dataset derived from such a simulation could serve as reference for the setting of FDG-PET for MTV delineation. We wished to identify the most important factors influencing delineation of the target on PET images, and to search the best way of dealing with those factors to optimize delineation of the target volume. We tried to express this relationship using a formula.

## Methods

### Simulation of PET Images

Considering the factors of tumor size, background, spatial resolution and pixel size of PET, 88704 virtual PET images were generated in simulation by programming with MATLAB (Mathworks, Natick, MA, USA).

### The Virtual Tumor in Simulation

#### Tumor

The features of the virtual tumor should represent the “real” tumor and be controlled readily. Most clinically encountered tumors (at least those at a relatively early stage) are spherical or oval, so the tumor volume can be calculated with a different tumor diameter. Hence, we set our virtual tumor to be spherical, and its diameter was set at 2 to 100 mm at 1-mm intervals, which produced a total volume of tumor of 99. Theoretical and experimental results already demonstrated that the standardized uptake value (SUV) of the tumor was independent of the level of PVE (25), so the FDG uptake (SUV) of all virtual tumors was set to 10 and distributed uniformly to simplify the calculation.

### Background

Real tumors inside a human body are surrounded by various types of normal/abnormal tissue in which the distribution of radiotracers create background activity or “noise”. To simulate this scenario, background activity was set at values of 0, 0.1, 0.2, 0.3, 0.4, 0.5, 0.6, and 0.7 times the SUV of the tumor, which represents a target: non-target ratio of “no background” situation, 10:1, 10:2, 10:3, 10:4, 10:5, 10:6, and 10:7, respectively.

### PET System in Simulation

Currently, the spatial resolution of PET equipment used in clinical situations is 4 to 9 mm. A dedicated computer program was written for PET-imaging simulation based on the principle of PET imaging and linear systems theory. PET imaging and reconstruction simulation made use of spatial resolution and pixel size.

### Spatial Resolution

Generally, the spatial resolution of a PET system is quantified with full width at half maximum (FWHM) for point source spread function. In our simulation, FWHM was set at 2 to 9 mm at 1-mm intervals. This introduced eight “PETs” of different performance, which covers almost all clinical PET scanners in present-day use.

### Pixel Size

The pixel size, to certain extent, might be related to spatial resolution. In total, 14 values were set for this study: FWHM/20, FWHM/18, FWHM/16, FWHM/14, FWHM/12, FWHM/10, FWHM/9, FWHM/8, FWHM/7, FWHM/6, FWHM/5, FWHM/4, FWHM/3, and FWHM/2.

### Image Analyses

#### Threshold

Due to the PVE, the tumor boundary is blurred and its size is not displayed clearly on the image. The automatic-threshold method based on tumor metabolism is considered the most objective method for delineating target volume. The threshold was the cutoff value (usually as a percentage of the pixel maximum uptake value) by which the boundary of MTV was recognized from three-dimensional PET images. Considering the background, the definition of the threshold was extended as follows:

Formula 1 $$\text{SUV}\;\text{in target boundary}=(\text{SU}V_{\max}-\text{SU}V_{\text{bg}})*\text{xx}\%+\text{SU}V_{\text{bg}}$$

where SUV_bg_ is the SUV in the background, SUV_max_ is the maximum SUV in the tumor image. xx% = (SUV in target boundary − SUV_bg_)/(SUV_max_ − SUV_bg_) = 11%, 12%……90%.

In the simulation, xx% was set from 11% to 90%, which resulted in a total of 80 target volumes for each PET image to determine the relationship between xx% and target volume. Hence, the target boundary within which the target volume was equal to the tumor volume could be ascertained.

The SUV in the target boundary and xx% were named the “absolute threshold” and “relative threshold”, respectively, when the target volume was equal to the tumor volume. The “relative threshold” is usually termed “threshold”.

Using Formula 1, the threshold was expressed using Formula 2:

Formula 2 $$\mathrm{SUV of Threshold}=(\mathrm{absolute of threshold}-\text{SU}V_{\text{bg}})/(\text{SU}V_{\max}-\text{SU}V_{\text{bg}})\cdot 100\%$$

In Formula 2, the target boundary meets the boundary inside, whereby the volume equals the real tumor volume.

### Determination of the Threshold

PET images were running virtually with all the variables mentioned above in acquisition and reconstruction procedures counted in the simulation in turn. Then, the resulting target volume (MTV) was compared with that of the virtual target. The effects of those changing variables were correlated and displayed on the corresponding chart, and the relationships between those variables were determined. On the basis of those data, a formula for the threshold calculation was determined.

### The Phantom Validation Experiments

Experiments were conducted under similar conditions to those in previous study (26). The facility used was Discovery PET/CT 690 (GE Healthcare) scanners. One NEMA image quality phantom (Biodex) of PET was used to simulate the clinical conditions in this trial. The ^18^F-NaF concentration in the background was 5.3 kBq/ml, while the ones in the spheres of diameter 37, 22, 17, and 10 mm were 8:1 as to that of the background to simulate tumors. PET images were acquired and reconstructed (192 × 192 matrix size, 3.65 mm × 3.65 mm × 3.27 mm voxel size). The reconstruction algorithms were Ordered Subset Expectation Maximization (OSEM) algorithm, 24 subsets, 2 iterations, and Gaussian post filter with FWHM 8.0 mm, and with Time-of-Flight (TOF) and Point Spread Function (PSF) technologies. The spatial resolution was 8 mm in the clinical conditions. Then, the target volumes were delineated by both the threshold and the radiation oncologists.

## Results

### Relationship Between xx% and Target Volume

**Figure 1:**
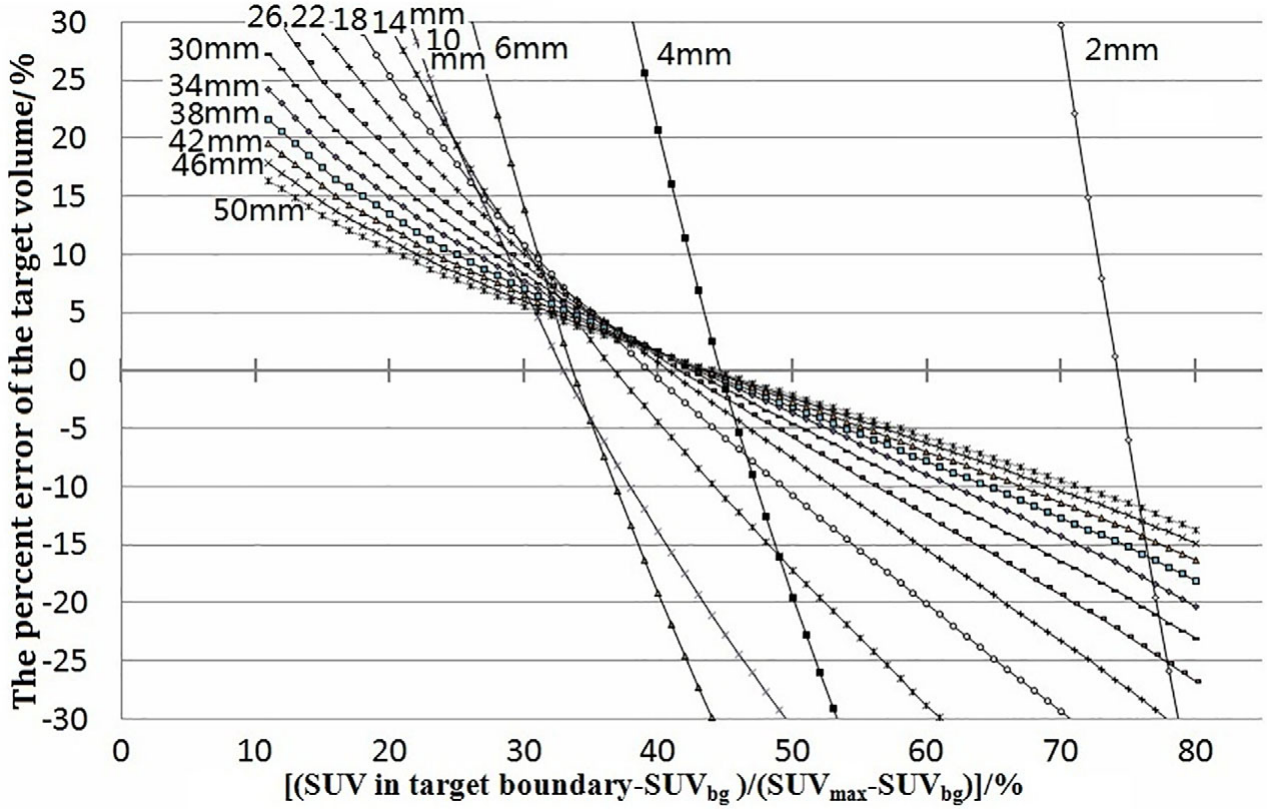
Percent errors of the target volume [(target volume − real volume)/real volume] were plotted against (SUV in target boundary − SUV_bg_)/(SUV_max_ − SUV_bg_). The junctions of curves with 0 error were the thresholds. They showed the error and how the threshold varied with tumor size. The labels 2–50 mm beside the curves were the diameters of the tumors.

In our simulation, with a fixed spatial resolution of PET, the relationship between xx% and the target volume varied with different tumor sizes. **Figure 1** shows the relationship between the percent error of the calculations of the target volume [(target volume − real tumor volume)/real tumor volume] and xx% for spherical tumors of different sizes. The simulation was carried out under a spatial resolution of 4-mm FWHM, tumor diameter of 2–100 mm, pixel size of 0.5 × 0.5 × 0.5 mm, and xx% of 11%–90% of SUV_max_. The intersections of each simulated curve with the horizontal axis at 0 represented the threshold for corresponding variables. The target volume delineated with the threshold equaled the real volume of the tumor. Some simulations using xx% of 11%–90% are shown in **Figure 1**.

### Relationship Between the Threshold and Tumor Size

**Figure 2:**
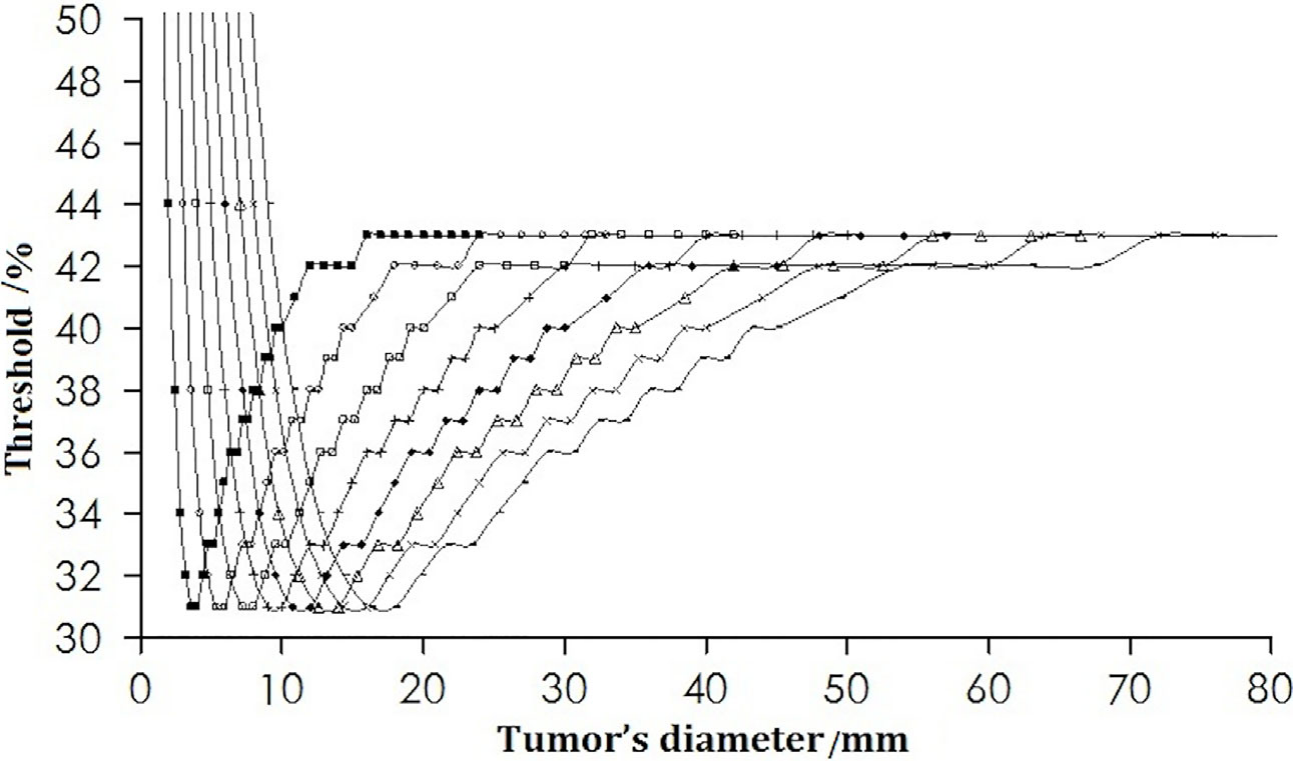
Change in the threshold with tumor diameter. The curves from left to right are for the spatial resolutions from 2 to 9 mm.

Under the condition of a fixed spatial resolution for PET, the thresholds corresponding to different tumor size were plotted (**Figure 2**). For tumors of all sizes in the simulation for a pixel size of 0.5 × 0.5 × 0.5 mm, the thresholds decreased initially to a minimum of 31% when the tumor diameter was twice the spatial resolution. This was followed by an increase as the diameter of the tumor increased further, reaching a maximum of 43%, and remained stable when the tumor size was 8 times the spatial resolution.

### Relationship Between the Threshold and Spatial Resolution

**Figure 3:**
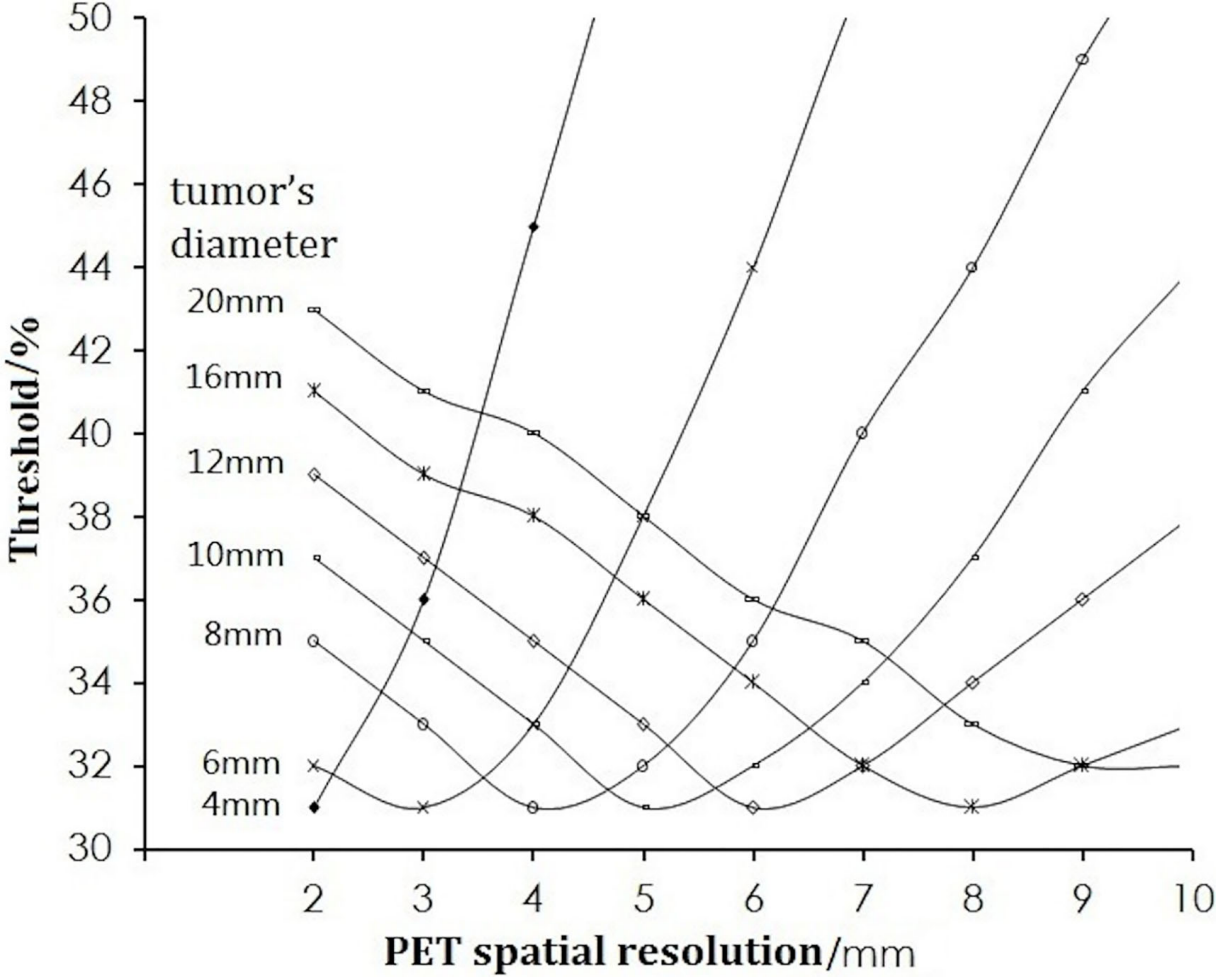
Change in the threshold with the spatial resolution.

For a certain volume of tumor, the thresholds are dependent upon the spatial resolution of the PET scanner. **Figure 3** shows that the threshold for different-sized tumors of pixel size 0.5 × 0.5 × 0.5 mm decreased as the spatial resolution worsened (increasing FWHM), dropped to a *nadir* of 31% at resolution (FWHM) = the half of the tumor diameter, and then increased with increasing FWHM.

**Figure 4:**
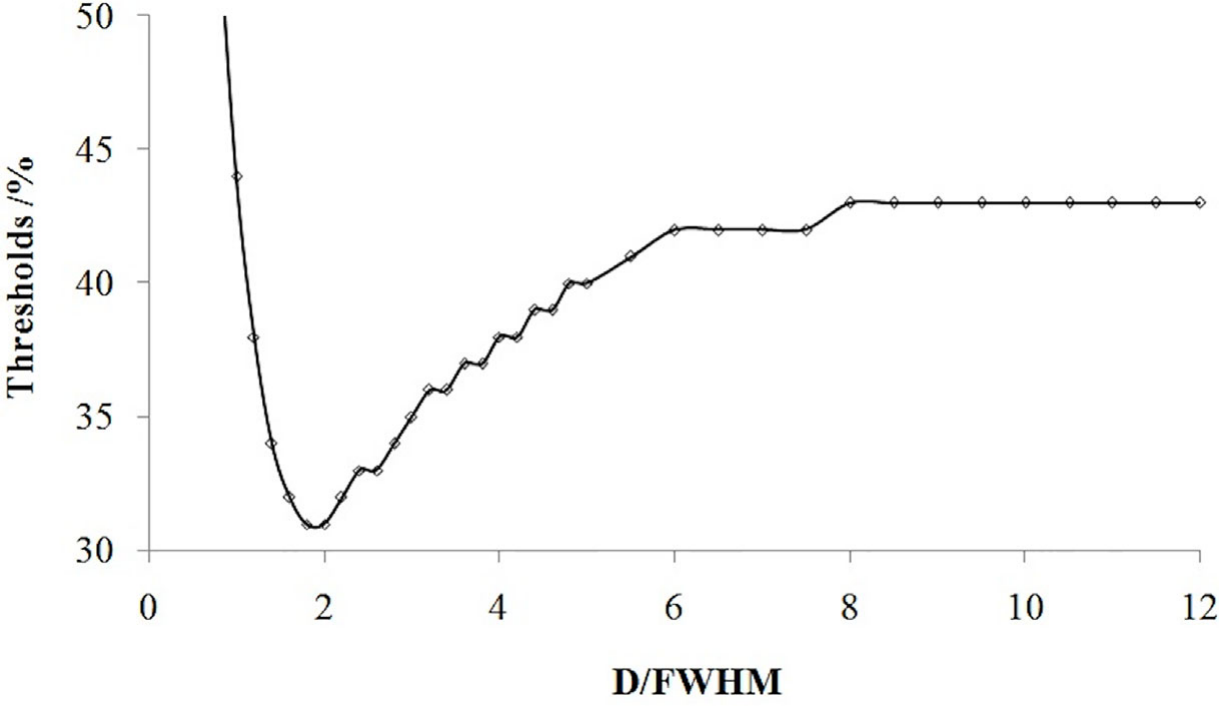
Relationship of the threshold and ratio of the tumor diameter (D) with the spatial resolution (FWHM).

If the tumor diameter was normalized to the spatial resolution (tumor diameter (D)/FWHM), all thresholds for all types of tumor diameters and spatial resolutions varied with D/FWHM in a unique way irrespective of tumor size or system resolution (**Figure 4**). The curves in **Figures 2** and **3** are summed as one curve in **Figure 4**.

**Table 1** lists some typical thresholds. From **Figure 4** and **Table 1**, the threshold decreased when D/FWHM was <2, reached a *nadir* when D/FWHM = 2, increased with increasing D/FWHM, and stabilized when D/FWHM >8.

**Table 1:** Some typical thresholds.

D/FWHM	Threshold/%
0.2	95
0.5	74
1	44
1.5	33
2	31
2.5	33
3	35
3.5	36
4	38
4.5	39
5	40
5.5	41
6	42
7	42
8	43
15	43

### Relationship Between the Threshold and Pixel Size of the Image

**Figure 5:**
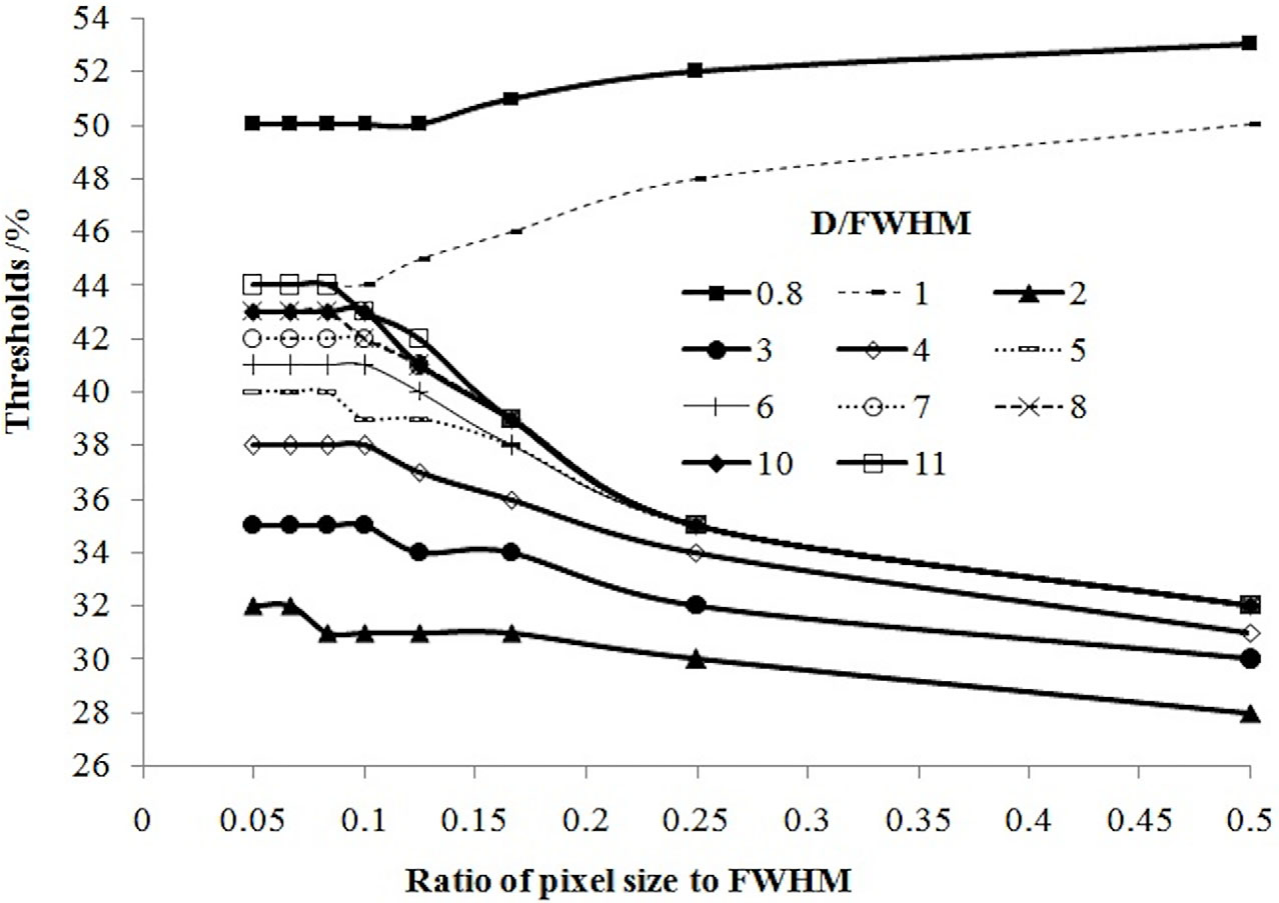
Variation in the threshold as a function of the pixel size normalized by FWHM.

In our simulation, the relationship between the threshold and pixel size was slightly complicated. **Figure 5** shows that if tumor size and pixel size were normalized by FWHM, the threshold was positively related to pixel size if D ≤ FWHM, or related negatively to pixel size if D > FWHM.

### Influence of the Background

The simulations mentioned above were applied to each background from SUV = 0 to SUV = 7. However, if the threshold mentioned above was [(absolute threshold − SUV_bg_)/(SUV_max_ – SUV_bg_) × 100%] in Formula 2 instead of [absolute threshold/SUV_max_], the threshold was independent of the background, but the absolute threshold changed with the background. According to Formula 2, the absolute threshold could be determined as Formula 3:

Formula 3 Absolute threshold=[(SUVmax−SUVbg)×threshold+SUVbg]

Formula 3 showed the influence of the background on the absolute threshold.

### Formula for Calculation of the Threshold

Considering the influencing factors of tumor size, spatial resolution, pixel size, and the background, the simulated image data were fitted to the formula for calculating the threshold.

Formula 4 $$D<2\;\text{FWHM}:\;\mathrm{threshold}=k_{1}{\left(D/\text{FWHM}\right)}^{-k2}$$

Formula 5 $$D\geq 2\text{FWHM}:\mathrm{Threshold}=c_{1}e^{-c_{2}\text{PS}/\text{FWHM}}(1-e^{-c_{3}D/\text{FWHM}\;e^{c_{4}\text{PS}/\text{FWHM}}})+C_{5}\text{PS}/\text{FWHM}$$

In Formula 4 and Formula 5, D is the tumor diameter (in mm), FWHM is the spatial resolution of PET (in mm) and PS is the pixel size (in mm). k1, k2, C1, C2, C3, C4, and C5 are dimensionless constants with fitted values of 46.57, 0.63, 50.568, 2.4758, 0.4617, 1.658, and 34.392, respectively. The fitting correlation coefficient R2 was 0.989 for Formula 4 (D < 2 FWHM) and 0.976 for Formula 5 (D ≥ 2 FWHM). The conditions for Formula 4 and Formula 5 were a spherical tumor of diameter 2−100 mm, a system spatial resolution FWHM = 2–9 mm, and pixel size = FWHM/20 – FWHM/2.

The threshold could be estimated using Formula 4 and Formula 5 for approximately spherical tumors, and then the absolute threshold could be calculated using Formula 3.

**Figure 6:**
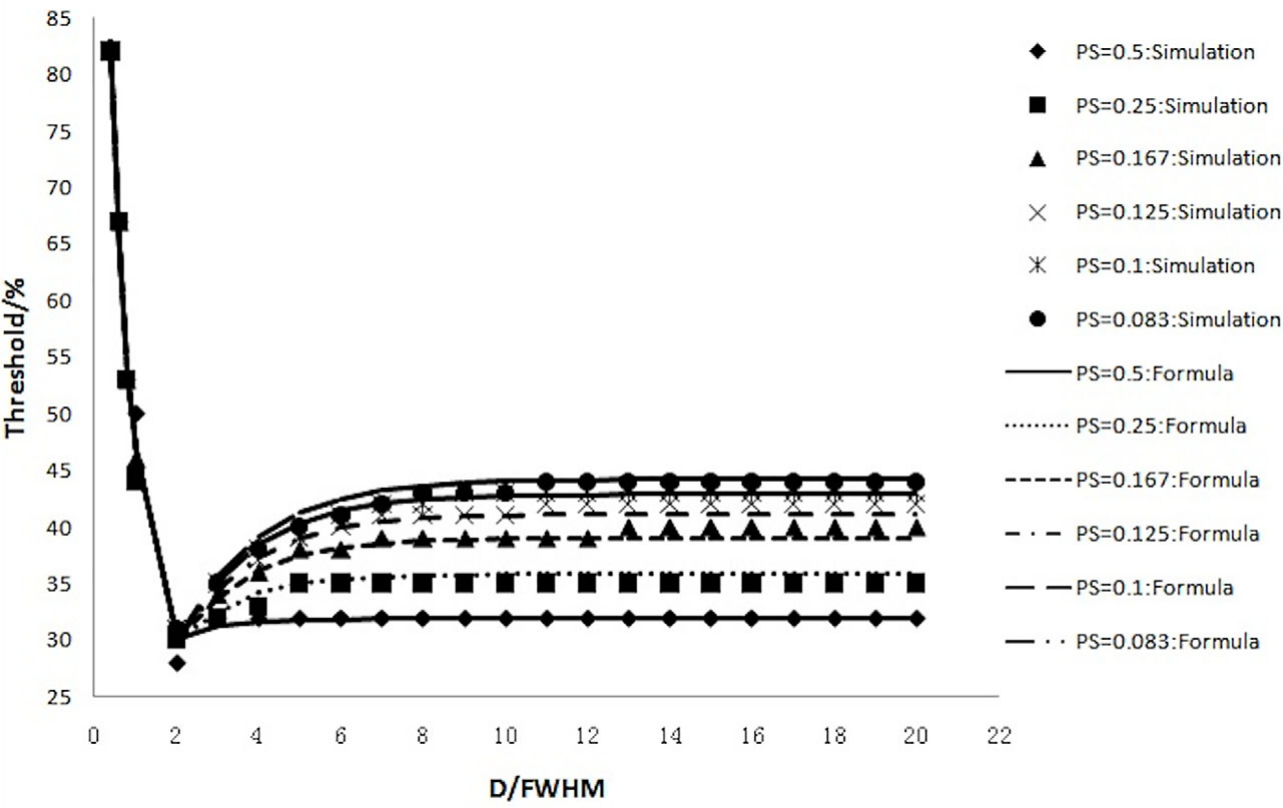
The thresholds calculated with the formula (smooth curve) and those obtained from simulation experiments and their variation with D/FWHM.

The accuracy of Formula 4 and Formula 5 was verified using simulation experimental data. The thresholds calculated with Formula 4 and Formula 5 were highly consistent with the values of simulation experiments (**Figure 6**). **Figure 6** shows that the thresholds calculated with the formula (smooth curve) and obtained from the simulation experiments varied with D/FWHM.

### The Phantom Validation Study

**Figure 7:**
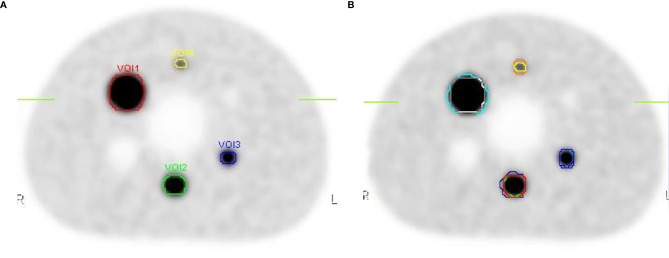
The contours on the image show the delineations done by the threshold and the physicians. **(A)** The target volumes delineated based on the threshold. **(B)** The target volume contoured by three radiation oncologists.

The threshold was calculated according to Formula 4 and Formula 5. Absolute threshold was calculated using Formula 3. Then, the target volumes were contoured based on Absolute threshold (**Figure 7A**), the calculated data are showed in **Table 2**. Meanwhile, three radiation oncologists contoured the target volume independently (**Figure 7B**). **Table 2** shows the average value of target volume performed by physicians. It can be seen clearly that the error of the target volume delineation that was calculated by the formula was less than 9%. The error of the volume contoured by radiation oncologists is much greater than that by threshold-based approach.

**Table 2:** Data analysis of the phantom validation experiment.

Inner diameter of hot spheres/mm	Volume of hot spheres/cm^3^	Thresholds calculated with the formula/%	Ratio of absolute thresholds to SUVmax/%	Target volume according to the thresholds/cm^3^	Relative deviation*/%	Target volume contoured by radiation oncologists/cm^3^			Relative deviation^†^/%		
						No. 1	No. 2	No. 3	No. 1	No. 2	No. 3
**37**	26.51	31.94	39	26.82	1.18	28.77	28.86	45.50	8.53	8.86	71.52
**22**	5.57	30.92	36	6.04	8.39	8.56	7.48	8.13	53.68	34.29	45.96
**17**	2.57	29.92	36	2.65	3.07	3.74	4.43	4.69	45.53	72.37	82.49
**10**	0.52	40.46	53	0.57	8.92	0.74	1.39	1.43	42.31	167.31	175.00

*The relative deviation was determined as the target volume according to the thresholds from that of its true volume.

^†^This relative deviation was determined as the target volume contoured by the radiation oncologists from that of its true volume.

## Discussion

Computer-simulation studies are used widely in medical imaging. Compared with real experiments, simulation studies offer advantages because they save research costs, obtain more data, and the experiment can be carried out under more controlled and normalized conditions without the influence of random factors. Because of the limited resolution of PET, a serious PVE occurs. Due to the PVE, the tumor boundary is blurred and its size cannot be obtained accurately on the image. We have studied the PVE and its correction for PET images of tumors (27). The present simulation study was undertaken to verify the factors that introduce errors in MTV measurement in PET imaging.

In the present study, PET imaging was simulated using different variables. The effects of those variables on the target volume were analyzed using a virtual spherical tumor taken as the standard of reality. Although the shapes of real tumors are varied, the heterogeneity of object shape and intra-tumor uptake were not taken into account in the formula. The main considerations are as follows. If we divide the tumor into the surface shell part and the inner part, we can find that the non-uniformity of the inner part is more serious. But the heterogeneity of inner part has no effect on the pixel value of the tumor boundary, and it has no effect on the results of this study. Most often, tumor cells in the shell have good blood supply and active proliferation, the activity concentration of FDG is relatively uniform. Therefore, the heterogeneity of tumor has limited influence on the threshold contour method to determine the boundary.

We showed that the delineating target threshold changed according to the tumor size, spatial resolution, background, and reconstruction pixel size. When the spatial resolution, background, and reconstruction pixel size were fixed, the threshold varied only with the tumor size, which corresponded to the clinical situation for delineation of targets for different-sized tumors. For the convenience of application, we used formulae to express this rule.

**Figures 1** and **2** revealed that the threshold changed with tumor size. The threshold increased sharply with decreasing tumor diameter when the tumor diameter was <2 FWHM, which was because the PVE is severe in smaller tumors. The threshold was the minimum at a tumor diameter equal to 2 FWHM and then increased as the tumor diameter increased further, reaching a maximum of 43% and remaining stable when the tumor size was 8 times the spatial resolution. For a PET system in clinical use with a resolution of 6-mm FWHM and background of 0, the threshold would be the minimum of 31% for a tumor of diameter <12 mm (2 FWHM), whereas the value could stabilize gradually to 44% if the tumor diameter was >36 mm (6 FWHM). The phantom studies of Jentzen and colleagues (7) showed that the threshold ranged from 36% to 44% for hot spheres with a volume >4 ml (diameter >2 cm), findings that are in accordance with our data. Okubo and colleagues and other researchers (6) showed that the threshold of a spherical tumor of diameter 22–37 mm ranged from 30% to 40%, findings that are in accordance with our data. Other scholars have shown that the threshold range is from 15% to 80%, but only several imaging situations were investigated in the studies mentioned above: their results were discrete. In this work, 88704 acquisitions covering all clinical situations were undertaken, and continual change of the threshold with tumor size was proposed. The reported discrete results (6, 7, 10–18) were covered in the change rule.

The threshold was also related to the spatial resolution (**Figure 3**). The threshold is different for different PET/computed tomography (CT) systems with different spatial resolutions, which could account for the different thresholds used by different scholars in their phantom studies over hot spheres of identical diameter. We found that if the tumor size was scaled by the spatial resolution, the threshold was no longer dependent on the resolution (**Figure 4**).

The influence of pixel size on the threshold is another important factor worthy of consideration (and one ignored by most scholars). In PET imaging, a target is displayed in an image matrix that is composed of pixels. Therefore, the clarity of the display and accuracy of the measurement of the target, to a certain extent, is related to the pixel size. The pixel size varies when using different image matrices and zoom. We showed that the threshold increased with pixel size for a tumor with a diameter less than the spatial resolution, and decreased with pixel size for a tumor with a diameter more than the spatial resolution (**Figure 5**).

Several scholars have conducted research on the influence of the background on the delineating threshold (6, 7, 14, 15), but a consensus has not been reached. Our results showed that changes in the absolute threshold with the background could be eliminated by using specific aspects of the relative threshold. The threshold based on Formula 2 was independent of the background, but this threshold was not the SUV at the best target boundary in the percentage of the maximum SUV. The SUV at the best target boundary could be calculated using Formula 3.

Based on the results stated above, two fitting formulae (4 and 5) were derived by which the thresholds could be calculated readily for the various sizes of tumors, different background and different spatial resolutions. These formulae considered various situations in clinical practice and could guide delineation of targets in different PET systems.

Delineation of the biologic target volume is dependent upon the threshold. Selection of the threshold to delineate the biologic target volume automatically is arbitrary and lacks theoretical and experimental bases. The influencing factors for the threshold were investigated systematically and two formulae (4 and 5) to calculate the threshold (using the tumor diameter, spatial resolution and pixel size) were derived in our study. The SUV at the best target boundary under any conditions could be calculated with Formula 4 and Formula 5, and automatic delineation for the biologic target volume could be achieved. The spatial resolution of PET and the reconstruction pixel size are given for a particular PET-CT facility and the tumor diameter can be obtained from CT images, so the threshold can be calculated using Formula 5. Delineation of the target volume with the threshold provides a reference value for delineating the biologic target volume objectively in clinical practice, which can be the starting point for radiotherapy.

In clinical practice, the contouring of target volumes is performed by radiation oncologists (28). Although detailed guidelines are used, the literature still showed poor repeatability, significant variability among radiation oncologists (29–31). It was also confirmed in our study. The threshold-based approach in this study was shown to delineate the gross tumor volume (GTV) accurately, which plays a crucial role in radiotherapy treatment planning. The main advantage of threshold-based tumor volume estimation was reduced necessity to manually identify the tumor boundary and would enhance the precision and objectivity of the target volume on PET images. However, for clinical practice, a validation study using real tumors should be carried out.

## Conclusions

The threshold to delineate the biologic target volume is related closely to the tumor diameter, spatial resolution of PET, and pixel size. A formula to calculate the threshold was derived based on the relationships. The threshold provides a reference value for delineating the biologic target volume objectively in clinical practice.

## Data Availability Statement

The original contributions presented in the study are included in the article/supplementary material; further inquiries can be directed to the corresponding author.

## Author Contributions

YC, JG, and FL designed research. JG, JZ, XZ, and BQ performed the imaging simulating experiment and collected data. JG and FL performed image analyses. YC and JT performed the statistical analyses. JG and FL wrote the paper. All authors contributed to the article and approved the submitted version.

## Funding

The work was funded by “China National Key Projects of Research and Development Grants (2016YFC0904600)” and “Grants from the International S&T Cooperation Program of China (2009DFA32960)”.

## Conflict of Interest

The authors declare that the research was conducted in the absence of any commercial or financial relationships that could be construed as a potential conflict of interest.

## Abbreviations

FWHM, full width at half maximum; MTV, metabolic tumor volume; PVE, partial volume effects.
